# Case Report: Clinical and pathological features of a metastasizing malignant mixed epithelial and stromal tumor of the kidney in a Siberian tiger (*Panthera tigris altaica*)

**DOI:** 10.3389/fvets.2025.1656594

**Published:** 2025-12-09

**Authors:** Tanja Švara, Mitja Gombač, Nika Kojc, Mojca Harej, Marjan Kastelic, Peter Kržan, Tamara Dolenšek, Pavel Kvapil

**Affiliations:** 1 Institute of Pathology, Wild Animals, Fish and Bees, Veterinary Faculty, University of Ljubljana, Ljubljana, Slovenia; 2 Institute of Pathology, Faculty of Medicine, University of Ljubljana, Ljubljana, Slovenia; 3 ZOO Ljubljana, Ljubljana, Slovenia; 4 Graduate student of the Veterinary Faculty, University of Ljubljana, Ljubljana, Slovenia

**Keywords:** tiger, kidney, tumor, mixed epithelial and stromal tumor, pathology

## Abstract

We describe a case of mixed epithelial and stromal tumor (MEST) of the kidney with widespread metastases in an eight-year-old male Siberian tiger (*Panthera tigris altaica*) from the ZOO Ljubljana. The tiger presented with hematuria, and after unsuccessful treatment attempts with antibiotics, an ultrasound examination followed by laparotomy was performed. During the laparotomy, a large encapsulated lesion weighing 9.3 kg was found in the location of the left kidney and surgically removed. Histopathological examination of the lesion revealed an encapsulated tumor with a biphasic growth pattern consisting of mesenchymal and epithelial components. Numerous mitoses, hemorrhages, and necrosis were found in the mesenchymal component, which resembled ovarian stroma, leading to the diagnosis of malignant MEST of the kidney. After 3 months, the tiger clinically deteriorated and was euthanized after diagnostic imaging revealed multiple metastases. At necropsy, numerous metastases were found at the site of the previously excised tumor, at the laparotomy scar, in the peritoneum, liver, pancreas, omentum, mesentery, lungs, and sternal lymph nodes. Microscopically, the tumor lesions consisted only of the malignant mesenchymal component. To our knowledge, this is the first report of malignant MEST of the kidney with peritoneal seeding and distant metastases in animals.

## 1 Introduction

Tumors in captive tigers are uncommon. Mostly single cases of tumors such as pancreatic neuroendocrine tumor (1), Sertoli cell tumor (2), mesothelioma (3, 4), malignant peripheral nerve sheath tumor (5), meningioma (6), maxillary epithelial odontogenic tumor (7), pancreatic adenocarcinoma and Brunner’s gland adenoma (8), mandibular squamous cell carcinoma (9), Leydig cell tumor (10), cutaneous metastatic melanoma (11) and mammary carcinoma (12) have been reported to date. In addition, there are some retrospective studies on tumors in captive wild felids, which also report the occurrence of tumors in tigers (13–17) with the most common being mammary adenocarcinomas (13, 15), uterine leiomyomas (14) and thyroid adenomas (14, 15).

Mixed epithelial and stromal tumor (MEST) of the kidney is a rare biphasic tumor consisting of epithelial and stromal components (18, 19). Due to its rarity and overlapping microscopic features, the tumor has been described under various names, such as adult mesoblastic nephroma, cystic hamartoma of the renal pelvis, leiomyomatous renal hamartoma, and more recently as renal epithelial and stromal tumor (REST) (20). According to data from human medicine, MEST are usually unilateral, confined to the kidney and renal pelvis (21) and occur predominantly in women (19, 22), typically during perimenopause (22). Microscopically, MEST show a biphasic growth pattern with cysts of different sizes and glands embedded in a spindle cell stroma that resembles ovarian stroma (22). Most MEST are benign tumors, but there are also rare cases with malignant transformation of the epithelial or stromal component (20, 23–30) and aggressive clinical behavior (30–34). Only a few cases have recurred (31, 32) or have either metastasized to the lymph nodes, liver and lungs or have metastasized systemically (22, 33, 35, 36).

To our knowledge, three reports of MEST have been published in veterinary medicine, in a ringtail lemur (*Lemur catta*) (37), a beagle dog (38) and a mouse (39). However, all cases exhibited benign clinicopathologic features and had not metastasized.

The aim of our study is to describe a case of malignant MEST in a Siberian tiger (*Panthera tigris altaica*) with very aggressive clinical behavior with peritoneal seeding and distant metastases that led to euthanasia 3 months after initial diagnosis based on excisional biopsy. We report the history, gross and histopathologic findings of the excisional biopsy, imaging results, and necropsy findings.

## 2 Case description

An eight-year-old intact male Siberian tiger (*Panthera tigris altaica*) weighing 180 kg from the ZOO Ljubljana was presented with hematuria. The animal showed no other clinical signs of disease and did not respond to initial antibiotic treatment for suspected urinary bladder infection. As the animal was not trained for voluntary ultrasound examination, it was performed under general anesthesia. During the examination, an abdominal mass was detected on ultrasonography, which warranted an immediate laparotomy during the same general anesthesia. Anesthesia was induced with 300 mg ketamine (Ketamidor 100 mg/mL, VetViva Richter GmbH, Austria), 15 mg midazolam (Midazolam Accord 5 mg/mL, Accord Healthcare, Poland), 10 mg butorphanol (Butomidor 10 mg/mL, VetViva Richter GmbH, Austria), and 8 mg medetomidine (Domitor 1 mg/mL, Orion Corporation, Finland), administered intramuscularly. The dosage of the drugs was based on standard species-specific protocols. Following induction of anesthesia, an intravenous catheter was placed and the tiger was intubated with an endotracheal tube. Despite apparent sedation, a positive palpebral reflex was noted, indicating insufficient anesthetic depth. Therefore, an additional bolus of ketamine was administered intravenously to deepen anesthesia. Anesthesia was maintained with isoflurane administered via anesthetic circuit. Before extubation, reversal agents were administered subcutaneously: atipamezole (Antisedan 5 mg/mL, Orion Corporation, Finland) to antagonize the effect of medetomidine, and flumazenil (Anexate 100 μg/mL, Cheplapharm Arzneimittel GmbH, Germany) to reverse the effect of midazolam. The laparotomy revealed a very large encapsulated lesion at the location of the left kidney, which was then surgically removed. During the same procedure, after inspection of the abdominal cavity, a splenectomy was performed due to a nodule on the spleen. No other lesions suggestive of metastases were found in the abdominal cavity and thoracic radiographs were unremarkable. The tiger made a full recovery, and hematologic and biochemical parameters were within normal limits on the day of surgery.

The entire encapsulated lesion and spleen were immediately chilled and submitted to the Institute of Pathology, Wild Animals, Fish and Bees, of the Veterinary Faculty University of Ljubljana.

**Figure 1:**
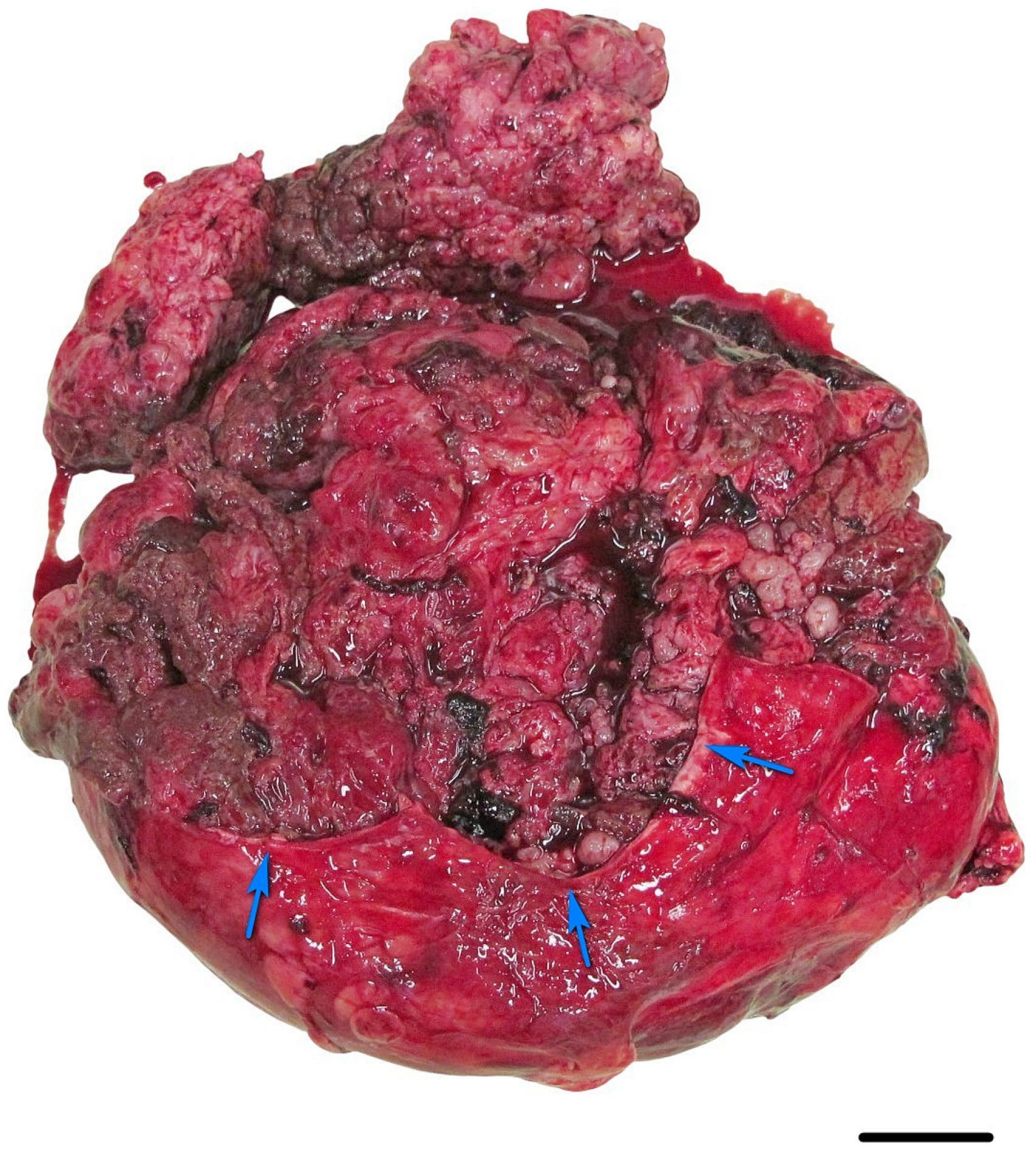
The excisional biopsy of the lesion in the left kidney of a Siberian tiger (*Panthera tigris altaica*), diagnosed with malignant mixed epithelial and stromal tumor (MEST). The well-circumscribed tumor was encapsulated by a grey-white capsule (blue arrows). The surface of the tumor after partial removal of the capsule was grey-red, lobulated and with solid and cystic areas. The solid areas displayed an arboriform architecture. Bar = 5 cm.

The lesion from the location of the left kidney measured 35 × 26 × 12 cm, weighed 9.3 kg, and was well circumscribed by a gray-white, 5-mm-thick capsule. After partial removal of the capsule, the lesion’s surface was gray-red, lobulated, and had both solid areas with an arboriform architecture and cystic areas (Figure 1). The cut surface of the lesion was gray-red, solid, cystic and bosselated with numerous hemorrhages and necrotic areas. The spleen had a well-demarcated, exophytic, nodular, gray-red lesion measuring 2 × 1.8 × 1.4 cm with a red cut surface.

Representative samples of both lesions were fixed in 10% neutral buffered formalin, routinely processed for microscopic examination, sectioned at 4 μm and stained with hematoxylin and eosin (H&E) and examined under a light microscope. Immunohistochemistry was performed according to the manufacturer’s instructions to confirm the origin of the neoplastic cells and to investigate a possible hormonal influence on tumor pathogenesis. The procedure was performed on selected 4-μm-thick paraffin sections. Details of the utilized primary antibodies and immunohistochemistry protocols used are provided in Table 1.

**Table 1:** Details of the primary antibodies and immunohistochemical protocols.

Primary antibody, clone, and catalogue number	Manufacturer	Antigen retrieval	Antibody dilution	Time and temperature of incubation of the primary antibody	Detection system	IHC staining procedure
Multicytokeratin, AE1/AE3, (NCL-L-AE1/AE3-601)	Novocastra, Germany	CC1, 64 min, 95 °C	1/50	32 min, 36 °C	Ventana, UltraView (Ventana Medical Systems Inc., Tucson, AZ, USA)	Automated
GATA3, L50-823, (390 M-16)	Cellmarque, USA	CC1, 32 min, 100 °C	1/50	32 min, 37 °C	Ventana, UltraView (Ventana Medical Systems Inc., Tucson, AZ, USA)	Automated
Estrogen receptors, SP1, (05278406001)	Ventana Roche, USA	CC1, 64 min, 95 °C	RTU	16 min, 36 °C	Ventana, UltraView (Ventana Medical Systems Inc., Tucson, AZ, USA)	Automated
Progesterone receptors, 1E2, (05277990001)	Ventana Roche, USA	CC1, 64 min, 95 °C	RTU	12 min, 36 °C	Ventana, UltraView (Ventana Medical Systems Inc., Tucson, AZ, USA)	Automated
CD31, JC70A, (M0823)	Dako Agilent, USA	CC1, 64 min, 95 °C	1/15	32 min, 36 °C	Ventana, UltraView (Ventana Medical Systems Inc., Tucson, AZ, USA)	Automated
CD34, QBEnd-10, (M0751)	Dako Agilent, USA	CC1, 64 min, 95 °C	1/20	32 min, 36 °C	Ventana, UltraView (Ventana Medical Systems Inc., Tucson, AZ, USA)	Automated
FVIII (poly), (A0082)	Dako Agilent, USA	/	1/800	32 min, 36 °C	Ventana, UltraView (Ventana Medical Systems Inc., Tucson, AZ, USA)	Automated
Desmin, DE-R-11, (05267005001)	Ventana Roche, USA	CC1, 64 min, 100 °C	RTU	20 min, 36 °C	Ventana, OptiView (Ventana Medical Systems Inc., Tucson, AZ, USA)	Automated
αSMA, 1A4, (202 M-96)	Cellmarque, USA	CC1, 64 min, 95 °C	1/100	32 min, 36 °C	Ventana, UltraView (Ventana Medical Systems Inc., Tucson, AZ, USA)	Automated
Calretinin, SP65, (05992184001)	Ventana Roche, USA	CC1, 56 min, 100 °C	RTU	20 min, 36 °C	Ventana, OptiView (Ventana Medical Systems Inc., Tucson, AZ, USA)	Automated
Vimentin, EPR3776, (ab92547)	Abcam, UK	CC1, 56 min, 100 °C	1/600	20 min, 36 °C	Ventana, OptiView (Ventana Medical Systems Inc., Tucson, AZ, USA)	Automated
CK20, SP33, (05587760001)	Ventana Roche, USA	CC1, 56 min, 100 °C	RTU	20 min, 36 °C	Ventana, OptiView (Ventana Medical Systems Inc., Tucson, AZ, USA)	Automated
Pax8, SP348, (ab227707)	Abcam, UK	CC1, 64 min, 100 °C	1/100	32 min, 36 °C	Ventana, OptiView (Ventana Medical Systems Inc., Tucson, AZ, USA)	Automated
CD10, 56C6, (NCL-L-CD10-270)	Novocastra, Germany	CC1, 56 min, 100 °C	1/15	32 min, 36 °C	Ventana, OptiView (Ventana Medical Systems Inc., Tucson, AZ, USA)	Automated
MelanA, A103, (05278350001)	Ventana Roche, USA	CC1, 72 min, 100 °C	RTU	32 min, 36 °C	Ventana, OptiView (Ventana Medical Systems Inc., Tucson, AZ, USA)	Automated
HMB45, Hmb45, (M0634)	Dako Agilent, USA	CC1, 64 min, 100 °C	1/20	32 min, 36 °C	Ventana, UltraView (Ventana Medical Systems Inc., Tucson, AZ, USA)	Automated
Carbon anchidrase (CAIX), EP161, (379R-14)	Cellmarque, USA	CC1, 56 min, 95 °C	1/100	20 min, 37 °C	Ventana, OptiView (Ventana Medical Systems Inc., Tucson, AZ, USA)	Automated
Uroplakin III, AU1, (696168)	Progen, Germany	Citrate buffer, pH 6.0, MW (1,100 W), 20 min	1/200	60 min, 23 °C	DAKO REAL™ EnVision Detection System Peroxidase/DAB+, Rabbit/Mouse (Dako, Denmark)	Manual
p63, (CM163A)	Biocare Medical, USA	Citrate buffer, pH 6.0, MW (1,100 W), 20 min	1/200	60 min, 23 °C	DAKO REAL™ EnVision Detection System Peroxidase/DAB+, Rabbit/Mouse (Dako, Denmark)	Manual

RTU, ready to use.

Microscopic examination of the lesion from the location of the left kidney revealed a multinodular tumor composed of epithelial and stromal components, the majority of which was well demarcated from the renal parenchyma by a fibrous capsule.

**Figure 2:**
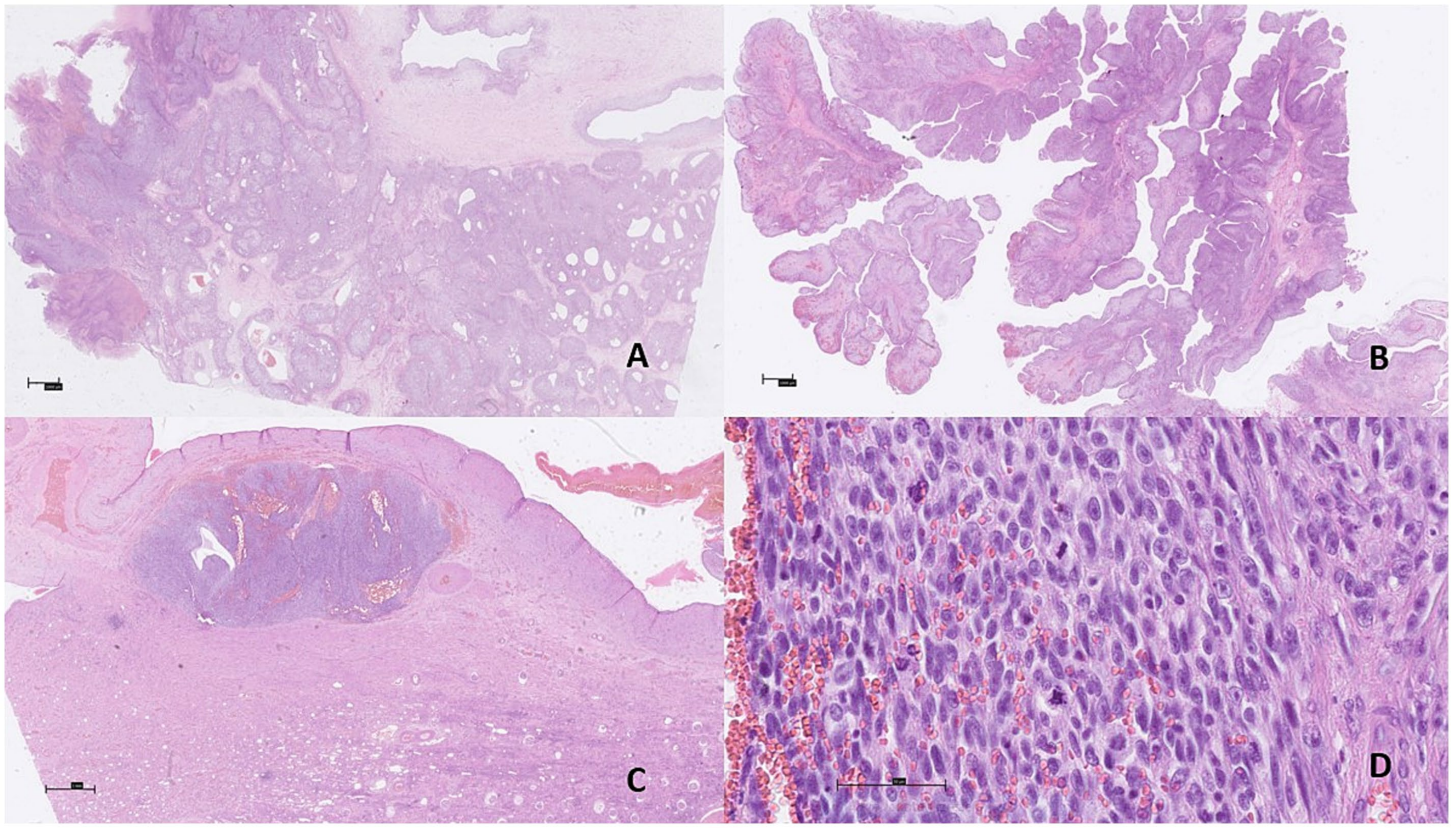
Microscopic findings of malignant mixed epithelial and stromal tumor (MEST) in the left kidney of a Siberian tiger (*Panthera tigris altaica*). The tumor was composed of epithelial and stromal components. **(A,B)** The epithelial component displayed papillary, tubular and cystic structures lined by epithelial cells. H&E, bar = 1,000 μm. **(C)** Focus of densely packed pleomorphic spindle stromal cells. H&E, bar = 1,000 μm. **(D)** High mitotic activity and atypical mitoses in the spindle stromal cells. H&E, bar = 50 μm.

**Figure 3:**
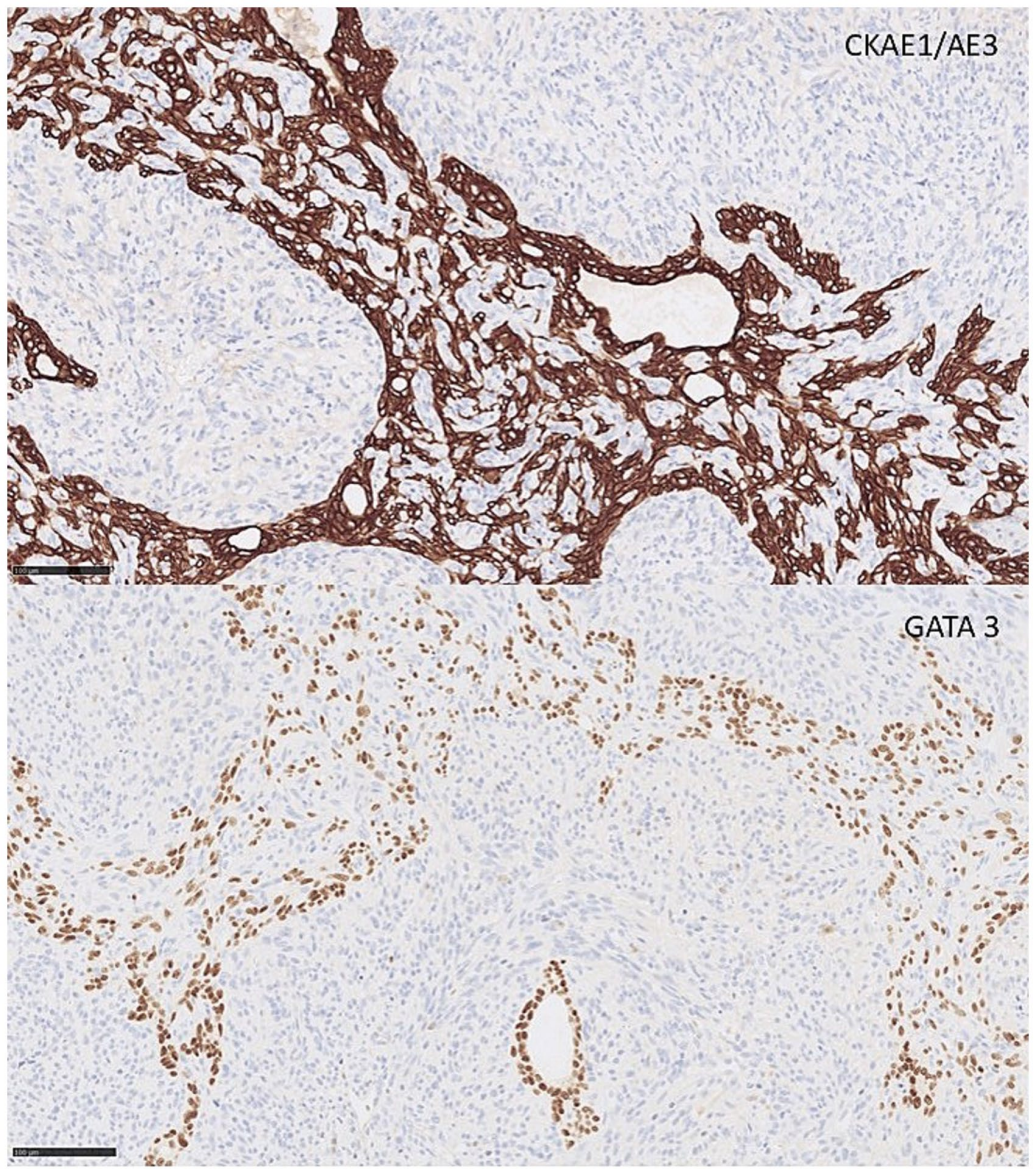
Microscopic findings of malignant mixed epithelial and stromal tumor (MEST) in the left kidney of a Siberian tiger (*Panthera tigris altaica*). Epithelial cells lining tubular structures were immunolabeled for GATA3 and CK AE1/AE3, while the stromal component showed immunolabeling for vimentin and αSMA. Immunohistochemistry, bar = 100 μm.

The epithelial component displayed tubular and cystic structures lined by cuboidal to columnar neoplastic epithelial cells resembling urothelium with umbrella cells, with the cystic spaces containing serous or proteinaceous fluid (Figures 2A,B). The neoplastic epithelial cells exhibited mild anisocytosis, had a small to moderate amount of pale eosinophilic cytoplasm, round to oval nuclei exhibiting moderate anisokaryosis, and a single prominent nucleolus or an inconspicuous nucleolus. Mitoses were rare, with 1 or less than 1 mitosis per 10 highpower field (2.37 mm^2^). The tubular structures exhibited positive immunolabeling for CKAE1/AE3, GATA3 and PAX8, while the stromal component was negative (Figure 3). Despite multiple attempts to optimize the protocol, no specific immunolabeling for estrogen and progesterone receptors, calretinin, CD20, p63, CD31, CD34 and uroplakin III was detected in either the tumor or the positive controls.

The stromal component consisted predominantly of neoplastic spindle cells reminiscent of ovarian stroma and multifocal groups of polygonal to round neoplastic stromal cells. The neoplastic spindle cells formed interwoven bundles and were multifocally condensed around neoplastic epithelial structures and blood vessels. In addition, multifocal papillary-like structures of neoplastic spindle cells were observed protruding into the lumina of the cystic structures and covered by neoplastic epithelial cells. The stromal cells exhibited moderate anisocytosis, with small to moderate amounts of eosinophilic cytoplasm. The nuclei exhibited moderate anisokaryosis, were large, oval to round with coarse chromatin, prominent nucleoli and frequent mitotic figures, 90 per 10 high-power fields (2.37 mm^2^), including atypical mitoses (Figures 2C,D). This component also demonstrated extensive necrosis and hemorrhage, with expansile rather than infiltrative tumor growth toward the surrounding renal tissue. Invasion of blood and lymphatic vessels was not observed. Stromal component expressed diffuse positive immunolabeling for CD10, vimentin, and desmin, and focal imunolabeling for alpha smooth muscle actin (αSMA), consistent with smooth muscle differentiation. Immunohistochemical stains for Melan-A, HMB-45, carbonic anhydrase IX (CAIX) and von Willebrand factor/factor VIII complex (FVIII) were negative in both the epithelial and stromal components. Interstitial fibrosis and tubular atrophy were observed in the remaining renal parenchyma.

Based on the gross, microscopic and immunohistochemical features of the tumor, a malignant MEST of the kidney was diagnosed. The nodular lesion in the spleen was diagnosed as hematoma.

After 3 months, the tiger clinically deteriorated. He developed lethargy and cachexia, with marked weight loss and muscle wasting despite normal food intake. Radiographic and ultrasound imaging revealed multiple metastases in the lungs and abdominal cavity. Based on the poor prognosis and in accordance with ethical and welfare considerations, the tiger was euthanized *ad tabulam* using 40 mL of T-61 (1 mL/ 5 kg) (1 mL containing embutramide 200 mg, mebezonium iodide 50 mg, and tetracaine hydrochloride 5 mg; Intervet International BV, Boxmeer, Netherlands) administered intravenously after it had already been under general anesthesia (combination of medetomidine (40 mcg/kg), ketamine, butorphanol and midazolam 0.1 mg/ kg) for follow-up diagnostic procedures, and immediately submitted for necropsy.

**Figure 4:**
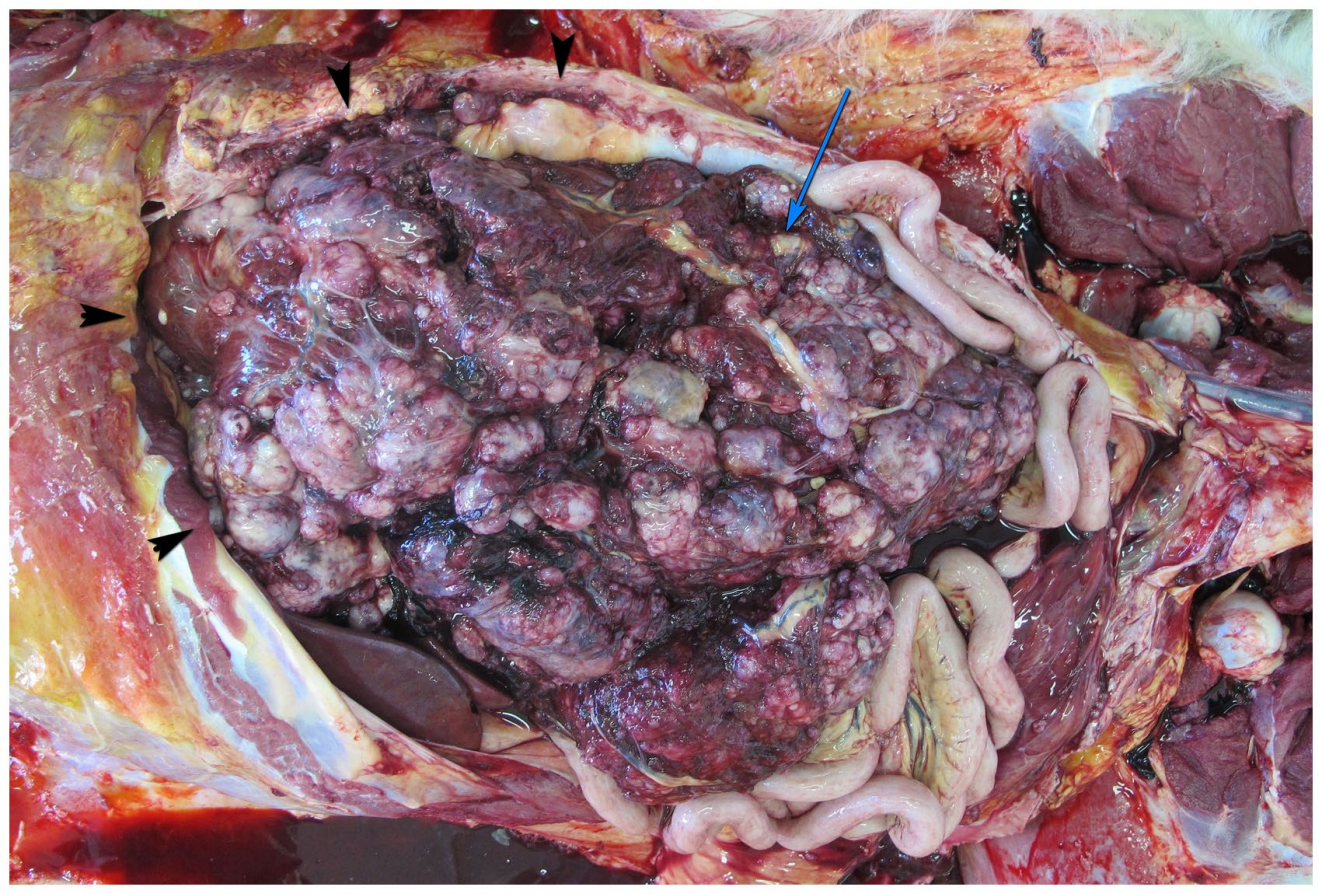
Necropsy findings in a Siberian tiger (*Panthera tigris altaica*) with malignant mixed epithelial and stromal tumor (MEST). Abdominal cavity with widespread peritoneal seeding. Tumors of different sizes are scattered in the abdominal cavity at the site of the previously excised tumor (blue arrow) and in the omentum. Arrowheads indicate the costal arch.

At necropsy, numerous tumors of various sizes were found at the site of the previously excised tumor of the left kidney, in the area of the laparotomy scar, in the parietal peritoneum, in the serosa of the stomach and urinary bladder, in the liver, pancreas, omentum and mesentery (Figure 4). The tumor at the site of the previously excised renal tumor was the largest and measured 25 × 17 × 16 cm, the tumor in the area of the laparotomy scar measured 18 × 9 × 3 cm, while the tumors in the parenchymal organs, the omentum and the mesentery measured up to 9 × 2.5 × 2 cm. The tumors were round to oval, multinodular, gray-red, smooth, poorly demarcated and firm, with red-gray, smooth, bosselated cut surfaces. These tumors and the finding of two liters of watery, slightly opaque fluid in the abdominal cavity were consistent with peritoneal seeding. Numerous tumors ranging in size from 2 mm to 10 × 6 × 6 cm, grossly resembling the tumors in the abdominal cavity, were also scattered throughout the lung lobes. The sternal lymph nodes were severely enlarged and measured up to 8 × 4 × 4 cm. The tiger was in poor body condition. Moderate eccentric hypertrophy of the left ventricle with mild epicardial fibrosis, moderate endocardial fibrosis and severe dilatation of the right ventricle were also noted. In addition, there was 0.5 liters of yellow serous fluid in the thoracic cavity.

Samples of tumors from all the above listed organs and sites as well as samples from the right kidney, intestine, adrenal glands, mesenteric lymph nodes, heart, and brain were fixed in 10% buffered formalin, routinely embedded in paraffin, sectioned at 4 μm, stained with H&E, and examined under a light microscope. Microscopically, the tumors were poorly demarcated, nonencapsulated, infiltrative, densely cellular, and consisted exclusively of a malignant mesenchymal (stromal) component that resembled the stromal component of the primary tumor of the left kidney. The mitoses were numerous and often atypical. Numerous necroses and hemorrhages were scattered throughout the tumors.

The case was diagnosed as malignant MEST of the left kidney with widespread peritoneal seeding and distant metastases of the mesenchymal (stromal) component of the tumor to the lungs and sternal lymph nodes.

## 3 Discussion

MEST are rare in humans and account for 0.2% of all renal tumors (30). Most MEST are benign, but in very rare cases malignant transformation occurs (23–30, 40) and aggressive clinical behavior has been described (30–34). MEST with benign histopathologic features but extension into the inferior vena cava has also been described (41).

The gross, microscopic and immunohistochemical features of the tumor described in this tiger closely resemble those described in other cases of malignant MEST in humans (20, 23–30, 34). The epithelial component showed positive imunolabeling for cytokeratins and GATA3, while expression of vimentin, αSMA, and desmin confirmed smooth muscle differentiation within the stromal component. Given the biphasic architecture, morphological features, and aggressive biological behavior, several malignant biphasic renal tumors were considered in the differential diagnosis. These included papillary renal cell carcinoma with prominent spindle cell stroma, sarcomatoid renal cell carcinoma, and synovial sarcoma. Additional biphasic neoplasms, such as mesothelioma, epithelioid hemangiosarcoma, and urothelial carcinoma, were also included in the differential based on tumor architecture and anatomic location.

Correlation of histopathological and immunohistochemical findings enabled exclusion of these differential diagnoses. The presence of a well-defined mesenchymal component lined by an epithelial component ruled out papillary renal cell carcinoma with spindle cell stroma. Renal cell carcinoma, including sarcomatoid variants, was excluded by the absence of CAIX expression. Epithelioid hemangiosarcoma was excluded by negative immunostaining for FVIII.

In malignant MEST in humans, malignant transformation can occur in either the epithelial or stromal component (20, 23–30), but most commonly malignant MEST has a malignant sarcomatous (stromal) component (25, 26, 29, 31, 32, 34), which is similar to our case. The malignant component in the herein described MEST resembled monophasic undifferentiated synovial sarcoma (23), synovial sarcoma (32), sarcoma with rhabdoid differentiation (24), rhabdomyosarcoma and chondrosarcoma (25), carcinosarcoma (36), undifferentiated sarcoma (26) and papillary renal cell carcinoma (28, 34). Sarcomas and carcinosarcomas typically lack expression of epithelial markers by immunohistochemistry. In contrast, the diffuse and strong immunolabeling for CK AE1/AE3 observed in the epithelial component is characteristic of MEST and differs from synovial sarcoma, in which cytokeratin expression is usually patchy, weak, and irregularly distributed among tumor cells (23, 25, 31, 32). PAX8, the tissue-specific transcription factors expressed primarily in the renal and Mullerian systems and also in Wolffian duct structures, has also been reported in MEST (20, 28, 41). Furthermore, coexpression of CD10 (20, 25, 36, 40), desmin (23, 36, 40), and αSMA (20, 23, 31, 40) in the stromal component provides additional support for the diagnosis of MEST.

In animals, MEST are even rarer than in humans. To the authors’ knowledge, only three cases have been described in animals, specifically in a 14.5-year-old female ringtail lemur, a 22 month-old female beagle dog, and a 32-week-old female mouse, all of which had only benign pathomorphologic features (37–39). All three cases reported in animals were diagnosed in females (37–39) and were considered young (38) or middle-aged (37, 39).

Despite the gross and microscopic similarities of the tumor diagnosed in the tiger, the case differs from human MEST in many other features.

Most human MEST are diagnosed in women (29, 34), with a female to male ratio of 6:1 to 10:1, in perimenopause and at an average age of about 45 years (19, 42). Some studies describing long-term oral estrogen therapy and immunopositivity for estrogen and progesterone receptors suggest a possible role of hormonal stimulation in the pathogenesis of MEST (42–44). Only a few cases of MEST have been described in male patients (26, 27, 40, 45, 46). The age of men with MEST is variable; according to some data, patients were older, with an average age of 71 years (47), while other cases of MEST were diagnosed in forty-year-olds (45, 46) or even in 19-year-old men with no history of hormone therapy (27). In some cases, a history of hormone therapy for prostate cancer has been described, which may have an impact on tumor progression (26).

The tiger was an eight-year-old intact male, which given the species’ average lifespan of 15 to.

20 years, corresponds to middle age (48). It was not under hormone therapy. Despite multiple attempts to optimize the protocol, immunolabeling of the tumor for estrogen and progesterone receptors was unsuccessful, thereby precluding assessment of their expression in the tumor. Due to the unsuccessful receptor immunolabeling, the involvement of hormonal mechanisms in the pathogenesis of the tumor cannot be confirmed, although this seems unlikely. The most common symptoms in human patients with MEST of the kidney are hematuria, flank pain, a palpable mass, or a urinary tract infection. Approximately 25% of cases of MEST are diagnosed incidentally (43, 44). In animals, the ringtail lemur with MEST showed reduced appetite and weight loss, and abdominal mass was palpated and visualized radiographically (37), while MEST in the dog and mouse were diagnosed incidentally at necropsy after the animals were euthanized for experimental studies (38, 39). The only clinical sign observed in the tiger prior to excisional biopsy and diagnosis of MEST of the kidney was hematuria that did not respond to antibiotic therapy.

The prognosis for MEST is generally good, while the prognosis for malignant MEST varies from case to case - some people are free of metastases or recurrence after nephrectomy, while some tumors have an aggressive clinical course with recurrence (27, 31, 32) or metastasize to the lymph nodes (22), liver (35) or lungs (33) or develop systemic metastases (36) and lead to death (26, 31, 32, 36). The shortest time from diagnosis to death was 5 months despite chemotherapy and radiotherapy (46). In our case, disease progression was also very rapid, with only 3 months from diagnosis to euthanasia due to widespread peritoneal seeding and metastases in the lungs and sternal lymph nodes. Complete resection with clean surgical margins is important for a good outcome in patients with malignant and benign MEST. Positive surgical margins, tumor disruption or tumor spillage during surgery may be associated with a poor outcome (32, 34, 46). Peritoneal seeding of benign MEST has been described after incomplete resection (48). Given the widespread peritoneal seeding and a large tumor in the abdominal wall at the laparotomy scar, we believe that the rapid progression of disease was probably the result of intraoperative tumor spillage and that the extreme size of the tumor probably contributed greatly to this outcome.

## 4 Conclusion

This is the first described case of malignant MEST of the kidney with peritoneal seeding and distant metastases in a Siberian tiger (*Panthera tigris altaica*) and the first such case ever reported in an animal. Although all previously reported MEST in animals have been benign and most MEST described in humans are also benign, the tumor described here showed microscopic features of malignant transformation with numerous and atypical mitoses, hemorrhages and necrosis on excisional biopsy. After only 3 months, the tiger was euthanized as its health had deteriorated considerably. This was the result of tumors at the site of the previously excised kidney tumor and laparotomy scar, peritoneal seeding, and metastases in the lungs and sternal lymph nodes, which microscopically consisted of the malignant component of the previously removed MEST of the kidney. This indicates that extreme caution is required when surgically removing these tumors, as intraoperative tumor spillage may lead to peritoneal seeding and distant metastases within a short period of time.

## Data Availability

The raw data supporting the conclusions of this article will be made available by the authors, without undue reservation.
